# The complete mitochondrial genome of *Ceutorhynchus obstrictus* (Marsham, 1802) (Coleoptera: Curculionidae)

**DOI:** 10.1080/23802359.2019.1667279

**Published:** 2019-09-19

**Authors:** Hyobin Lee, Jonghyun Park, Jieun Lee, Ki-Jeong Hong, Jongsun Park, Wonhoon Lee

**Affiliations:** a Department of Plant Medicine, Gyeongsang National University, Jinju, Republic of Korea;; b InfoBoss Co., Ltd, Seolleung-ro, Seoul, Gangnam-gu, Republic of Korea;; c InfoBoss Research Center, Seolleung-ro, Seoul, Gangnam-gu, Republic of Korea;; d Department of Plant Medicine, Sunchon National University, Sunchon, Korea;; e Institute of Agriculture and Life Science, Gyeongsang National University, Jinju, Republic of Korea

**Keywords:** *Ceutorhynchus obstrictus*, mitochondrial genome, phylogenetic relationship, Curculionidae, Korea

## Abstract

*Ceutorhynchus obstrictus* (Marsham, 1802) is a serious pest of oilseed rape (*Brassica napus* L.) in Europe and the USA. We have determined a 20,124 bp mitogenome of *C. obstrictus* which includes 13 protein-coding genes, 2 ribosomal RNA genes, 22 transfer RNAs, and a single large non-coding region of 2,773 bp. The base composition was AT-biased (81.4%). Hypothetical ORFs are identified in the control region. Phylogenetic trees present that *C. oibstricus* is clustered with *Alcides juglans* (Alcidinae). It also shows polyphyletic manner for two tribes, requiring more mitogenomes to resolve it.

*Ceutorhynchus obstrictus* (Marsham, 1802), also known as cabbage seedpod weevil, is a major pest of oilseed rape (*Brassica napus*) originated in Europe. It has successfully settled across the globe including the U.S. (Cárcamo et al. 2001). It poses a major threat to economic sustainability of canola production in western Canada (Cárcamo et al. 2001). In 1995, *C. obstrictus* was first collected from Gimhae region, Korea, consequently became a serious insect pest on *B. napus* in Korea (Kim et al. 2018). To understand its genetic background, we determined its complete mitogenome as first mitogenome in Ceutorhynchinae.

Genomic DNA of *C. obstrictus* collected from Seogwipo-si, Jeju-do in Korea in 2019 (33°52′50ʺN, 126°93′04ʺE; specimen is stored in Gyeongsang National University, Korea, Accession number: Coll#HB002) was extracted using DNeasy Brood & Tissue Kit (QIAGEN, Hilden, Germany). HiSeqX was used for sequencing (Macrogen Inc., Seoul, Korea). Filtering, *de novo* assembly, and gap-filling processes were done by Velvet 1.2.10 (Zerbino and Birney 2008), Trimmomatic 0.33 (Bolger et al. 2014), SOAPGapCloser 1.12 (Zhao et al. 2011), BWA 0.7.17 (Li 2013), and SAM tools 1.9 (Li et al. 2009). Geneious R11 11.1.5 (Biomatters Ltd, Auckland, New Zealand) and ARWEN (Laslett and Canbäck 2008) were used to annotate mitogenome of *C. obstrictus* based on *Eucryptorhynchus brandti* mitogenome (Nan et al. 2016).

*Cryptopone obstrictus* mitogenome (MN180050) is 20,124 bp and GC ratio is 18.6%. It contains 13 protein-coding genes (PCGs), 2 rRNAs, and 22 tRNAs. Range of tRNA size is 64–71 bp, which is smaller than those of some insect species, such as *Aiolocaria hexaspilota* (55–70 bp; Seo et al. 2019) and *Cryptopone sauteri* (56–78 bp; Park, Kwon, Park, 2019). Gene order of *C. obstrictus* is similar to mitogenomes of other weevils, which is the ancestral gene order of all insects. Interestingly, inside the control region, there are two hypothetical ORFs of which directions are reversed in the same coordination and amino acids are different. No homologous genes of them are found in non-redundant database. Mitogenome of *Scolytinae* sp. (KX035192) also shows the same phenomenon. In addition, mitogenomes of *Anisandrus dispar* (NC_036293), *Curculionidae* sp. (KX035176), and *Hypothenemus* sp. (KX035163) also have a hypothetical ORF in control region, indicating not exceptional case. It may be explained by expansion of fungal mitogenomes of genus *Aspergillus* (Xu et al. 2018; Park et al. 2019).

We inferred the phylogenetic relationship of 38 Curculionid species including *C. obstrictus* and two outgroup species of *Cyllorhynchites ursulus* (MH156809) and *Platystomos albinus* (KX087337). Multiple sequence alignment was conducted by MAFFT 7.388 (Katoh and Standley 2013) using concatenated alignments of all PCGs. Bootstrapped maximum likelihood and neighbor-joining trees were constructed using MEGA X (Kumar et al. 2018). *Ceutorhynchus obstrictus* (Ceutorhynchinae) was clustered with *Alcides juglans* (Alcidinae) with relatively low bootstrap value in both trees (Figure 1), indicating that more mitogenomes in both subfamilies are required to unravel their phylogenetic relationship. Phylogenetic trees present paraphyletic manners in two subfamilies, Cryptorhynchinae and Entiminae, with relative low bootstrap values (Figure 1). Our mitogenome will provide phylogenetic insights in Curculionidae family in near future.

**Figure 1.**
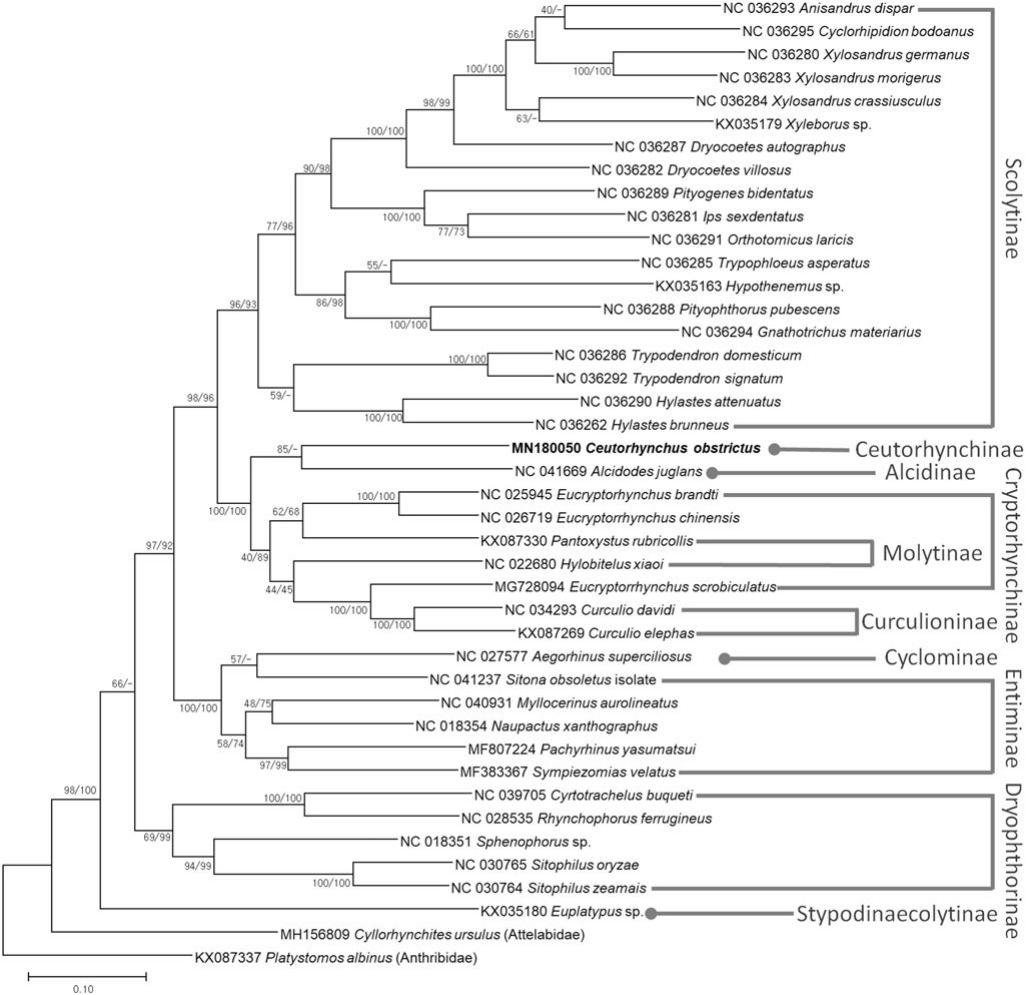
Maximum likelihood (bootstrap repeat is 1000)and neighbor-joining (bootstrap repeat is 10,000) phylogenetic tree of 38 Curculionidae species: *Ceutorhynchus obstrictus* (MN180050: this study), *Anisandrus dispar* (NC 036293), *Cyclorhipidion bodoanus* (NC 036295), *Xylosandrus germanus* (NC 036280), *Xylosandrus morigerus* (NC 036283), *Xylosandrus crassiusculus* (NC 036284), *Xyleborus* sp. (KX035179), *Dryocoetes autographus* (NC 036287), *Dryocoetes villosus* (NC 036282), *Pityogenes bidentatus* (NC 036289), *Ips sexdentatus* (NC 036281), *Orthotomicus laricis* (NC 036291), *Trypophloeus asperatus* (NC 036285), *Hypothenemus* sp. (KX035163), *Pityophthorus pubescens* (NC 036288), *Gnathotrichus materiarius* (NC 036294), *Trypodendron domesticum* (NC 036286), *Trypodendron signatum* (NC 036292), *Hylastes attenuatus* (NC 036290), *Hylastes brunneus* (NC 036262), *Alcidodes juglans* (NC 041669), *Eucryptorhynchus brandti* (NC 025945), *Eucryptorrhynchus chinensis* (NC 026719), *Pantoxystus rubricollis* (KX087330), *Hylobitelus xiaoi* (NC 022680), *Eucryptorrhynchus scrobiculatus* (MG728094), *Curculio davidi* (NC 034293), *Curculio elephas* (KX087269), *Aegorhinus superciliosus* (NC 027577), *Sitona obsoletus* (NC 041237), *Myllocerinus aurolineatus* (NC 040931), *Naupactus xanthographus* (NC 018354), *Pachyrhinus yasumatsui* (MF807224), *Sympiezomias velatus* (MF383367), *Cyrtotrachelus buqueti* (NC 039705), *Rhynchophorus ferrugineus* (NC 028535), *Sphenophorus* sp. (NC 018351), *Sitophilus oryzae* (NC 030765), *Sitophilus zeamais* (NC 030764), *Euplatypus* sp. (KX035180), and two outgroup species: *Cyllorhynchites ursulus* (MH156809, Attelabidae), *Platystomos albinus* (KX087337, Anthribidae). Phylogenetic tree was drawn based on maximum likelihood phylogenetic tree. The numbers above branches indicate bootstrap support values of maximum likelihood and neighbor joining phylogenetic trees, respectively.
